# Centre for Advanced Research on Addictive Behaviors: a national framework for digital wellness and youth mental health targeting behavioral addictions in India

**DOI:** 10.3389/fpsyt.2025.1747027

**Published:** 2026-03-12

**Authors:** Yatan Pal Singh Balhara, Rajeev Ranjan, Ragul Ganesh, Siddharth Sarkar, Shivanand Kattimani, Vandana Saxena, Subhash Das, Vishal Dhiman, Swarndeep Singh, Rachna Bhargava, Yumnam Surbala Devi, Tanmay Joshi, Anindo Majumdar, Syed Hube Ali

**Affiliations:** 1 Behavioral Addictions Clinic (BAC) and Centre for Advanced Research on Addictive Behaviours (CAR-AB), National Drug Dependence Treatment Centre (NDDTC), All India Institute of Medical Sciences (AIIMS), New Delhi, India; 2 All India Institute of Medical Sciences, Patna, India; 3 Jawaharlal Institute of Postgraduate Medical Education & Research (JIPMER) Puducherry, Puducherry, India; 4 AIIMS, New Delhi, India; 5 Central Institute of Education (CIE), Department of Education, University of Delhi, Delhi, India; 6 Department of Psychiatry, North Eastern Indira Gandhi Regional Institute of Health & Medical Sciences (NEIGRIHMS), Shillong, India; 7 All India Institute of Medical Sciences, Rishikesh, India; 8 Vardhman Mahavir Medical College (VMMC) & Safdarjung Hospital, Delhi, India; 9 College of Nursing, AIIMS, New Delhi, India; 10 All India Institute of Medical Sciences, Bhopal, India; 11 UNICEF, New Delhi, India

**Keywords:** addictive behaviors, artificial intelligence, digital wellness, gambling, gaming, prevention and intervention, public health research, shopping/buying

## Abstract

Digital wellness and digital technology related addictions have emerged as critical public health research priorities in India, especially given the growing impact of behavioral addiction on youth mental health. The Centre for Advanced Research on Addictive Behaviours (CAR-AB) aims to promote safe digital technology use and enhance digital well-being among Indian youth. CAR-AB was conceptualized at All India Institute of Medical Sciences (AIIMS), New Delhi, in collaboration with leaders from health, public health, technology and education sectors and with the funding support from the Indian Council of Medical Research (ICMR). CAR-AB aims to establish a scientific, systematic, and sustainable framework for addressing addictive behaviors and promoting digital and overall well-being among Indian youth. CAR-AB is intended to develop and evaluate AI-based predictive models; validated intervention packages; training and capacity-building toolkit; national resource center on addictive behaviors; and policy and programmatic recommendations. CAR-AB envisions “Digital Wellness for All” by promoting safe and healthy use of digital technology.

## Introduction

Digital technologies have transformed modern life by reshaping communication, education, commerce, and recreation. India has been at the forefront of this digital revolution. In addition, it is also home to one of the world’s largest youth populations. While the benefits of technological expansion are undeniable, the excessive or problematic use of digital technology has become an emerging public-health crisis (1, 2).

For the purposes of this manuscript, behavioral addictions refer to maladaptive patterns of engagement in non-substance-related behaviors (e.g., gaming, gambling, internet use, social media use, smartphone use) characterized by impaired control, persistence despite negative consequences, and functional impairment, consistent with contemporary diagnostic frameworks such as ICD-11 and DSM-5 (3, 4).

Problematic use of digital technology refers to using technology in a pattern that leads to impaired control, increasing priority given to the behavior, and continued engagement despite negative consequences. It is associated with significant distress, dysfunction or both. It may or may not meet the diagnostic threshold currently. This can manifest in the form of use of digital devices (online and offline) and the internet. It can occur in various contexts including use of the internet, smartphones, gaming, social media, gambling, shopping/buying, Over the Top (OTT) content watching, pornography watching, and excessive screen time. These are all potentially addictive behaviors. While many of these have not been listed as specified diagnostic categories, ICD-11 makes provision for listing these under the residual categories in the section on addictive behaviors (5).

Digital wellness refers to a state of balanced, intentional, and developmentally appropriate use of digital technologies that supports psychological well-being, physical health, social functioning, and academic or occupational performance, while minimizing risk of harm (2).

Addictive behaviors associated with the use of the internet and digital devices are a growing public health concern, as reflected in multiple global systematic reviews and meta-analyses documenting increasing prevalence and associated harms across age groups (6, 7). These include, but are not limited to, problematic use of the internet, smartphone, gaming, social media, gambling, Over the Top (OTT) platform content, pornography, shopping/buying and screen time. Excessive or problematic engagement is associated with increased levels of stress, anxiety, depression and increased risk of addiction (8–12).

Global and Indian studies consistently demonstrate a substantial burden of problematic digital-technology use among youth. Meta-analyses report pooled prevalence estimates ranging from approximately 14–27% across internet, smartphone, and social media addictions, with comparable or higher rates reported in Indian school and college populations (6, 13, 14). Evidence from the COVID-19 period suggests further amplification of these behaviors (7). Youth have been reported to be at an increased risk of developing behavioral addictions (15–18). Collectively, these findings underscore the public-health relevance of problematic technology use among children, adolescents, and young adults.

The World Health Organization (WHO) formally recognized gaming disorder and gambling disorder as diagnosable conditions in the International Classification of Diseases, 11th Revision (ICD-11, 3). The DSM-5 previously identified addictive behaviors as a distinct category of disorders (4).

Globally there has been an increasing interest in addictive behaviors. This is reflected in the published literature on this theme over the past couple of decades (19–22). Previous research has demonstrated that those with high Internet addiction have lower scores of quality of life than those who were normal Internet users (23). Association between symptoms of gaming disorder and depression has been reported (24). Research on the interface of addictive behaviors and depression has explored multiple dimensions including cognitive distortion, insomnia, loneliness, self-esteem, social support, alexithymia, cybervictimization and academic performance (8–10). Research among children and adolescents has also reported that excessive gaming or social media use contributes to adverse consequences on their mental well-being (9). Internet addiction has been found to be positively correlated with alcohol use, smoking and an increased risk of suicidal behavior among adolescents (10). Previous research has also suggested the role of multiple determinants of addictive behaviors (25–29). Decrease in real life social community participation and academic achievement, and relationship problems have been found to be negative correlates of social networking sites usage (30). Earlier studies have also reported the neurobiological underpinnings of additive behaviors (31). The effects of internet addiction were seen throughout multiple neural networks. This included a mix of increases or decreases in functional connectivity in the default mode network, an overall decrease in functional connectivity in the executive control network, and no clear increase or decrease in functional connectivity within the salience network and reward pathway (32).

Much of the existing evidence linking excessive or problematic use of digital technologies with adverse mental-health outcomes is derived from cross-sectional observational studies. While these studies have been instrumental in documenting prevalence and associations, they are inherently limited in their ability to establish temporal sequencing or causal pathways. As a result, risks of reverse causality as well as residual confounding from unmeasured social, developmental, or contextual factors, cannot be ruled out.

## Research, policy and service gaps

Despite a growing body of literature, the predominance of cross-sectional designs has constrained understanding of developmental trajectories, mechanisms of risk, and points of intervention for problematic technology use among youth. The limited use of longitudinal designs and mediation-focused analyses has restricted the field’s ability to disentangle directionality and to identify modifiable pathways linking technology use with mental-health outcomes.

Existing evidence demonstrates high prevalence of problematic technology use and associated mental-health risks among Indian youth, as documented in national and international studies. The planned innovations under the CAR-AB initiative seek to address identified gaps through the development of predictive models, prevention-focused interventions, and implementation frameworks, which will be empirically tested in subsequent phases.

The rise of behavioral addictions parallels the rapid proliferation of digital technology. The introduction of smartphones in 2007 catalyzed exponential growth in online engagement, with India reporting over 750 million mobile internet users in 2025 (33). Excessive use of digital platforms has been linked to multiple psychiatric and psychosocial outcomes (8–10, 23). The problem is particularly concerning among children, adolescents and young adults, whose neurocognitive development renders them vulnerable to compulsive reward-seeking and emotional dysregulation (34). A clear interest in problematic use of digital technology among Asian countries has been reported (35).

Our earlier research explored various dimensions of addictive behaviors among the youth in India. These included prevalence and patterns of problematic internet use and gaming (36, 37); risk factors, determinants, and correlates; defining problematic thresholds such as internet-use duration or diagnostic criteria; other conceptual issues (38, 39); phenomenology and emerging forms of behavioral addiction, public health, media, and socio-legal perspectives, conceptual misnomers in defining behavioral addictions (1), COVID-19 consequences (40); neurophysiological study, and pertinent legal issues (41).

In a study of urban adolescents in India, Saikia et al. reported that internet addiction was strongly associated with depression (OR = 14, 95% CI = 7.9-24.6), stress (OR = 12, 95% CI = 5.5-25.7), and anxiety (OR = 3.3, 95% CI = 1.9-5.6), indicating substantially higher odds of these conditions among adolescents with problematic internet use compared to their peers. All reported associations were statistically significant (p<0.05), underscoring the clinical relevance of these findings (12). Sedgwick et al. reported adjusted odds ratios between 1.03 and 5.10 for suicide attempts among adolescents with high social-media engagement (42).

The consequences of addictive behaviors extend beyond health to include economic and social repercussions. The Economic Survey of India *2024–25* explicitly linked the rise in adolescent mental-health issues to overuse of internet and social media, calling for “school- and family-level interventions towards keeping children and adolescents away from the internet and improving mental well-being” (33).

Despite growing evidence of harm, India currently lacks coordinated, evidence-based policies and service frameworks addressing problematic technology use and digital wellness among youth, particularly within educational settings. Existing responses remain fragmented, underscoring the need for integrated, scalable approaches spanning health, education, and policy sectors.

In addition, India’s policy and service infrastructure lack dedicated mechanisms for promoting digital-wellness promotion or prevention and management of behavioral addictions. This gap has been highlighted in national policy analyses and expert commentaries, which note the absence of structured school-based or health-system–integrated digital wellness programs (33, 43, 44). Despite a large section of the population at-risk being in educational institutions (schools and colleges), there is a dearth of policies, plans, or programs that intend to address these issues in a systematic way. This assumes even greater relevance in context of the widespread advocacy for digitalization of education across all stages in school that puts the young learners at the interface of digital technology more often and for extended periods of time. Despite increasing digitalization of education under NEP 2020, contextually informed guidelines and implementation frameworks for safe technology use among children and adolescents remain limited (45). This necessitates the development of evidence-based standardized digital-wellness training curriculum for multiple stakeholders in the schools and colleges, including the leadership, teachers, parents, and, of course, the students.

The regulation of online gaming and gambling in India remains fragmented. Also, despite calls to have contextually relevant guidelines on potentially addictive behaviors and screen time, limited progress has been made (43).

While there has been interest in behavioral addiction in the country the research is still in its infancy (44). India still lacks a comprehensive, validated predictive model for early identification of at-risk youth; structured prevention-oriented interventions integrating mental health and education sectors; evidence-based clinical or school protocols for detection, referral, and management; standardized capacity-building programs for teachers, parents, and healthcare workers; and Policy documents addressing “digital wellness” within national mental-health strategies.

In response, the Centre for Advanced Research on Addictive Behaviors (CAR-AB) was conceptualized at All India Institute of Medical Sciences (AIIMS), New Delhi, in collaboration with leaders from health, public health, technology and education sectors and with the funding support from the Indian Council of Medical Research (ICMR). CAR-AB aims to establish a scientific, systematic, and sustainable framework for addressing addictive behaviors and promoting digital and overall well-being among Indian youth.

This manuscript is intended as a perspective and programmatic framework paper describing the rationale, conceptual underpinnings, objectives, and planned research activities of the CAR-AB. At this stage, no empirical results or outcome data are presented.

All outcomes, tools, intervention packages, predictive models, and policy impacts described in this article are planned or anticipated outputs of the CAR-AB initiative and have not yet undergone empirical validation. The methods outlined reflect the proposed study designs and implementation strategies that will be employed across multiple interrelated studies.

Empirical findings arising from individual studies conducted under the CAR-AB initiative (including predictive model performance, intervention effectiveness, and implementation outcomes) will be reported separately through dedicated protocol papers and results manuscripts. This paper therefore aims to delineate the scope, scientific rationale, and public-health relevance of the initiative, while clearly distinguishing planned research from completed analyses.

Throughout this manuscript, established findings from the existing literature are distinguished from planned research activities and anticipated outputs of the CAR-AB initiative. Descriptions of predictive models, interventions, training modules, and policy impacts refer to intended or proposed components that are yet to be empirically tested or validated. Empirical evaluation and impact assessment of these components will be reported separately following completion of the planned studies.

## Conceptual framework

**Figure 1:**
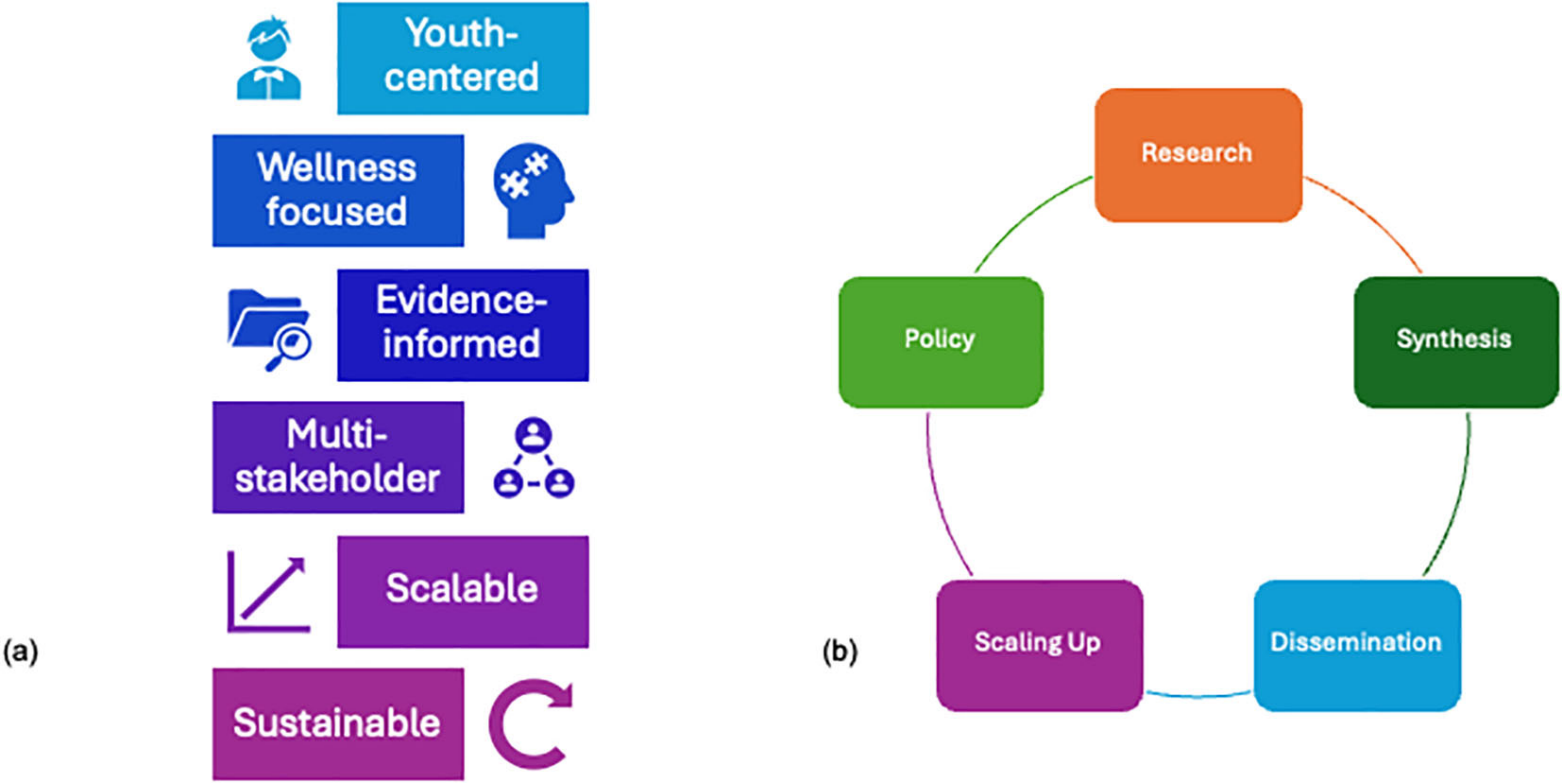
**(a)** Conceptual schematic framework for Centre for Advanced Research on Addictive Behaviours (CAR_AB) and **(b)** How CAR-AB envisions to take research findings to guide policy change.

CAR- AB is proposed as a youth- centered, wellness focused, evidence- informed, multi- stakeholder, scalable and sustainable initiative. In addition, it envisions that the research findings shall guide the policy change (**Figure 1**).

CAR-AB adopts an implementation- focused research paradigm, bridging the gap between research and practice. The Centre’s conceptual foundation integrates four interdependent pillars including scientific innovation (applying AI and behavioral-science methodologies to identify risk patterns and test scalable interventions); public health integration (aligning with national programs such as the National Mental Health Programme (NMHP), National Education Policy (NEP 2020), Digital India, etc.); transdisciplinary collaboration (engaging psychiatry, psychology, public health, education, and technology experts); and capacity and policy translation (establishing a National Resource Centre on Addictive Behaviors to inform curriculum, clinical guidelines, and digital-wellness policy).

**Figure 2:**
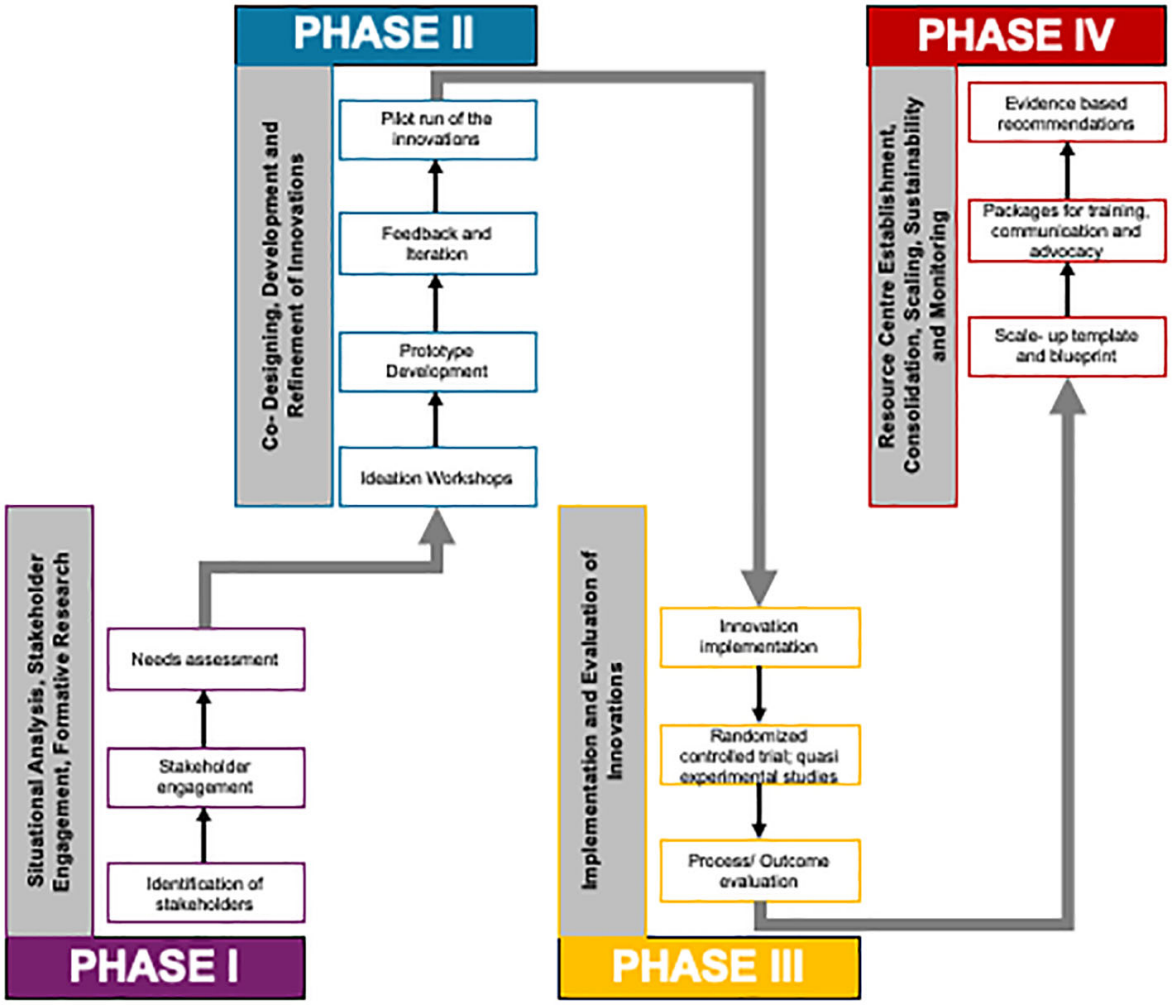
Conceptual schematic of various phases of Centre for Advanced Research on Addictive Behaviours (CAR-AB).

The initiative shall be rolled out using a phased approach. Since multiple studies shall be carried out as part of the initiative, the timelines for the phases of individual studies shall vary. A schematic representation of the various phases of the CAR- AB and the set of initial studies has been presented in **Figure 2**.

### Objectives

The primary objectives of CAR-AB are to:

• To develop and test AI- based predictive models to detect vulnerability to excessive or problematic use of technology guided by demographic, psychological, cognitive, behavioral, socio- environmental and digital phenotype correlates among the youth (children, adolescent, young adults).
• To develop and validate a comprehensive package of prevention- focused interventions targeted at youth (children, adolescents, young adults), parents and teachers for excessive or problematic use of technology and associated stress, depression, and anxiety among the youth (children, adolescent, young adults).
• To develop and test the effectiveness of a Screening, Brief Intervention and Referral to Treatment (SBIRT) package aimed at early detection and intervention for excessive or problematic use of technology and mitigate the associated stress, depression, and anxiety among the youth
• To develop and evaluate the standardized training modules to strengthen the capacity of the education and health sector workforce on the promotion of digital well- being among the youth
• To generate evidence-based recommendations on the promotion of digital well- being and the prevention of excessive or problematic use of the internet and technology and associated stress, depression, and anxiety among the youth

### Methods

The present manuscript acknowledges the limitations of the existing literature. This recognition has directly informed the design of the CAR-AB. By integrating comprehensive assessment, mediation and moderation analyses, and multi-site validation, the initiative is intended to advance the field beyond descriptive associations toward a more mechanistic and implementation-relevant understanding of behavioral addictions in youth. In addition, it aims to develop both therapeutic and prevention-focused interventions. Finally, by developing the resource material and guidelines it aims to extend the benefits of the initiative beyond the project sites.

To begin with, CAR-AB encompasses five interrelated studies. The overall design combines cross-sectional observational, qualitative formative, quasi-experimental, and randomized controlled trial designs and intervention methodologies. The studies shall be conducted across multiple sites. Relevant evaluation and analysis strategies shall be used for each study. The details of these studies shall be published as study protocols at a later stage. Full protocols will be published separately as protocol papers. A brief description of the studies is presented here.

**Figure 3:**
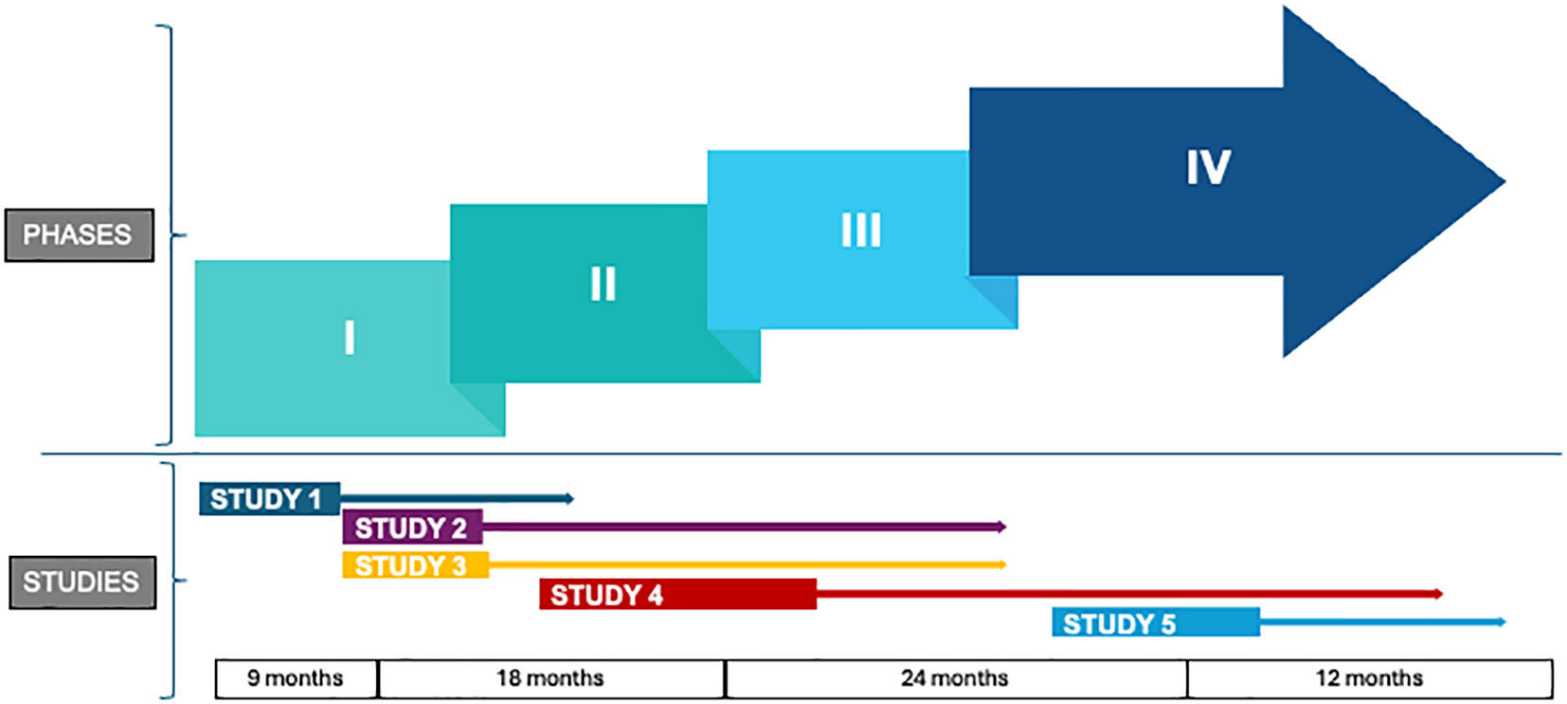
Conceptual; schematic of various phases and timeline for in the initial set of studies proposed as part of Centre of Advanced Research on Addictive Behaviours (CAR-AB). The time line of the phases shall vary for each study.

The proposed timeline for the roll out of various studies is presented in **Figure 3**.

Study 1: Creating AI- based predictive models to detect vulnerability to excessive or problematic use of technology.

Study 1 is a multi-site cross-sectional observational study conducted in schools (grades 7- 12) and colleges across six geographically diverse regions of India, designed to develop AI-based predictive models for vulnerability to excessive or problematic use of digital technologies and associated stress, anxiety, depression, and addiction risk. An indicative minimum sample size of approximately 1,080 participants has been planned, comprising both school and college students, based on prior Indian prevalence estimates to ensure adequate precision for estimating outcomes of interest and sufficient statistical power for machine-learning model development. The survey will capture diverse correlates including demographic, psychological, cognitive, behavioral, socio-environmental and digital phenotype. The participants will complete a fixed set of validated tools (e.g., WHO-5, PSS-4, TIPI, SAS-SV, 6-item IAT, IGDS9-SF, BSMAS, MULTICAGE CAD-4, BBGS, insomnia screens, and short cognitive tasks).Primary outcomes include validated measures of problematic technology use across multiple domains and associated psychological distress indicators. Model validation will involve internal train–test splits, cross-validation procedures, and assessment of discrimination, calibration, and error metrics, with cross-site comparisons undertaken to examine robustness and generalizability across regions. Detailed methods and analytic plans will be reported in a separate protocol publication.

Study 2: Development and validation of a comprehensive package of prevention- focused interventions targeted at youth, parents and teachers for excessive or problematic use of technology and associated stress, depression, and anxiety among the youth.

Study 2 will use an exploratory qualitative design and a framework-driven approach to build a prevention package for youth, parents, and teachers. Inputs will come from desk review, focus groups, and in-depth interviews with youth, parents, teachers, mental-health staff, public-health staff, and policy actors. The aim is to define the key risks, map the drivers, set clear targets for change, and select the most useful behavior-change tools. An expert panel will review all inputs and shape the final package. It will draw on behavior-change, learning, and social-cognition models and covers digital skills, emotion skills, safe use of devices, urge control, and help-seeking. A mixed-methods phase will test change in knowledge, skill, and confidence with pre- and post-measures. The development phase uses exploratory qualitative methods, including focus group discussions and in-depth interviews, with sample sizes guided by data-saturation principles across stakeholder groups. The validation phase adopts a quasi-experimental mixed-methods approach, with indicative sample sizes powered to detect medium effect sizes in pre–post evaluations of intervention impact. Validation will be conducted across multiple sites to assess feasibility, acceptability, and consistency of effects in diverse educational settings. A full protocol detailing intervention development and evaluation methods will be published separately.

Study 3: Development and test the effectiveness of a Screening, Brief Intervention and Referral to Treatment (SBIRT) package aimed at early detection and intervention for excessive or problematic use of technology and mitigate the associated stress, depression, and anxiety among the youth.

Study 3 evaluates a Screening, Brief Intervention, and Referral to Treatment (SBIRT) package using a multi-site randomized controlled trial design in educational settings, with linkage to health-care services for referral. Sample size calculations are based on detecting clinically meaningful reductions in problematic technology use and associated stress, depression, and anxiety, with allowances for attrition. The SBIRT package shall include screening tools for problematic use of digital technology and addictive behaviors, a brief intervention targeted at these problematic behaviors and a mechanism to link the persons with problematic patterns with appropriate health care services. Primary outcomes include behavioral and readiness changes, change in pattern of use, and uptake of referral pathways. Effectiveness will be assessed through between-group comparisons. This interventional study will be prospectively registered, and a detailed protocol will be published separately.

Study 4: Development and evaluation of standardized training modules to strengthen the capacity of the education and health sector workforce on the promotion of digital well- being among the youth.

Study 4 is a quasi-experimental mixed-methods study designed to develop and evaluate standardized training modules aimed at strengthening the capacity of education and health-sector personnel to promote digital well-being among youth. Indicative sample sizes are planned to detect moderate improvements in knowledge, skills, and confidence following training, with representation across participating regions and professional groups. Primary outcomes include pre–post changes in knowledge of problematic technology use, early identification skills, and self-reported preparedness to support youth digital well-being. Cross-site testing will assess scalability, contextual adaptability, and implementation feasibility. The full study protocol will be reported separately.

Study 5: Development of evidence-based recommendations on the promotion of digital well- being and the prevention of excessive or problematic use of the internet and technology and associated stress, depression, and anxiety among the youth.

Study 5 focuses on the development of evidence-based recommendations for digital-wellness promotion and prevention of excessive or problematic technology use by synthesizing findings from Studies 1–4. This study relies on systematic evidence integration, structured expert consultations, and stakeholder feedback. Primary outputs include policy-relevant recommendations, implementation frameworks, and scalable templates for integration into education and health systems. Cross-site applicability will be assessed through comparative synthesis of findings across regions and contexts. The methodological approach for this synthesis will be detailed in a separate protocol publication.

These studies have been summarized in **Table 1**.

**Table 1:** Overview of CAR-AB studies.

Study	Primary aim	Study design	Population	Setting/sites
Study 1	Develop AI-based predictive models for vulnerability to problematic technology use	Multi-site cross-sectional observational study	School students (≥12 years) and college students	Schools and colleges across six regions in India
Study 2	Develop and validate prevention-focused intervention packages	Exploratory qualitative plus quasi-experimental mixed-methods	Youth, parents, teachers, mental-health and public-health experts	Schools and colleges across multiple sites
Study 3	Test effectiveness of SBIRT package	Multi-site randomized controlled trial	Youth screening positive for problematic technology use	Educational settings with health-sector linkage
Study 4	Build capacity of education and health-sector workforce	Quasi-experimental mixed-methods	Teachers, counsellors, health professionals	Educational and health institutions
Study 5	Develop evidence-based recommendations and implementation frameworks	Evidence synthesis and expert consultation	Policymakers, experts, stakeholders	Multi-sectoral, national scope

This manuscript provides a high-level overview of planned methodologies and does not present empirical results. Full study protocols for the studies will be published separately, and interventional studies, including randomized and quasi-experimental designs, will be prospectively registered with appropriate national trial registries prior to implementation.

These studies will be supplemented with additional studies based on the needs and learning as the initiative rolls out.

The methodological descriptions presented here outline planned study designs and evaluation strategies. At the time of manuscript submission, the CAR-AB initiative is in the preparatory and phased initiation stage; central ethics approval has been obtained, site-specific approvals are secured, and participant recruitment and data collection has commended (for study 1) and not yet commenced (for Studies 2-5) across all sites and studies. Accordingly, descriptions of outcomes, validation, and impact reflect intended evaluation approaches rather than completed analyses.

### Ethical considerations

The CAR-AB initiative is intended to adhere to nationally and institutionally approved ethical standards, with informed consent (and assent for minors) and site-specific ethics approvals obtained prior to implementation. The protocol has received Institutional Ethics Committee (IEC) approval at AIIMS, New Delhi (Ref. No. IEC/2025/04/07). Approval shall be obtained from each of the participating sites as well. Participation will be voluntary, with informed consent (and parental consent and assent for minors). Data confidentiality will be maintained. The trials conducted as part of the studies shall be registered with the Clinical Trial Registry of India.

The planned studies emphasize minimizing participant burden and avoiding stigmatization, particularly when assessing vulnerability to problematic technology use.

Equity considerations inform the multi-site design, which is intended to enhance inclusivity by engaging youth from diverse geographic, socio-economic, and educational contexts. Study tools and interventions are designed to be developmentally appropriate and context-sensitive, with stakeholder engagement supporting relevance and acceptability across settings.

Given the planned use of digital phenotyping and AI-based analytics, data-protection and governance safeguards are prioritized. Data collection is intended to follow principles of minimization, anonymization, and secure storage, with transparency in model development supported through explainable analytic approaches and institutional oversight mechanisms. Developing predictive models in domains such as youth behavior raises certain ethical concerns. These include data privacy, potential stigmatization, and algorithmic bias. In this study, use of data minimization, anonymization, and interpretability mechanisms shall be incorporated to ensure transparency and explainability. This is in keeping with international recommendations for ethical deployment of AI in mental health (46).

### Expected Outcomes

CAR-AB will lead to the development of resources targeted at various stakeholders including youth, parents, teachers, counsellors/healthcare providers and policymakers and program managers. CAR-AB aims to deliver the following key outputs:

• AI-based predictive model: Development and empirical evaluation of an AI-based algorithm intended to enable early identification of vulnerability to problematic technology use and associated stress, anxiety, depression, and addiction risk among youth.
• Validated intervention packages: Development and testing of promotive, preventive, and SBIRT intervention modules, designed for use in schools, colleges, and community settings, with effectiveness to be empirically assessed as part of the CAR-AB studies.
• Training and capacity-building toolkit: Development and evaluation of standardized digital-wellness training curricula for educators, psychologists, and healthcare professionals.
• National resource center on addictive behaviors: Establishment of a national resource hub intended to support research coordination, data sharing, advocacy, and policy guidance on addictive behaviors.
• Policy and programmatic recommendations: Generation of evidence-informed recommendations intended to support integration of digital-wellness strategies into national programs such as the NMHP, NEP 2020, and child, adolescent, and general health initiatives.

**Figure 4:**
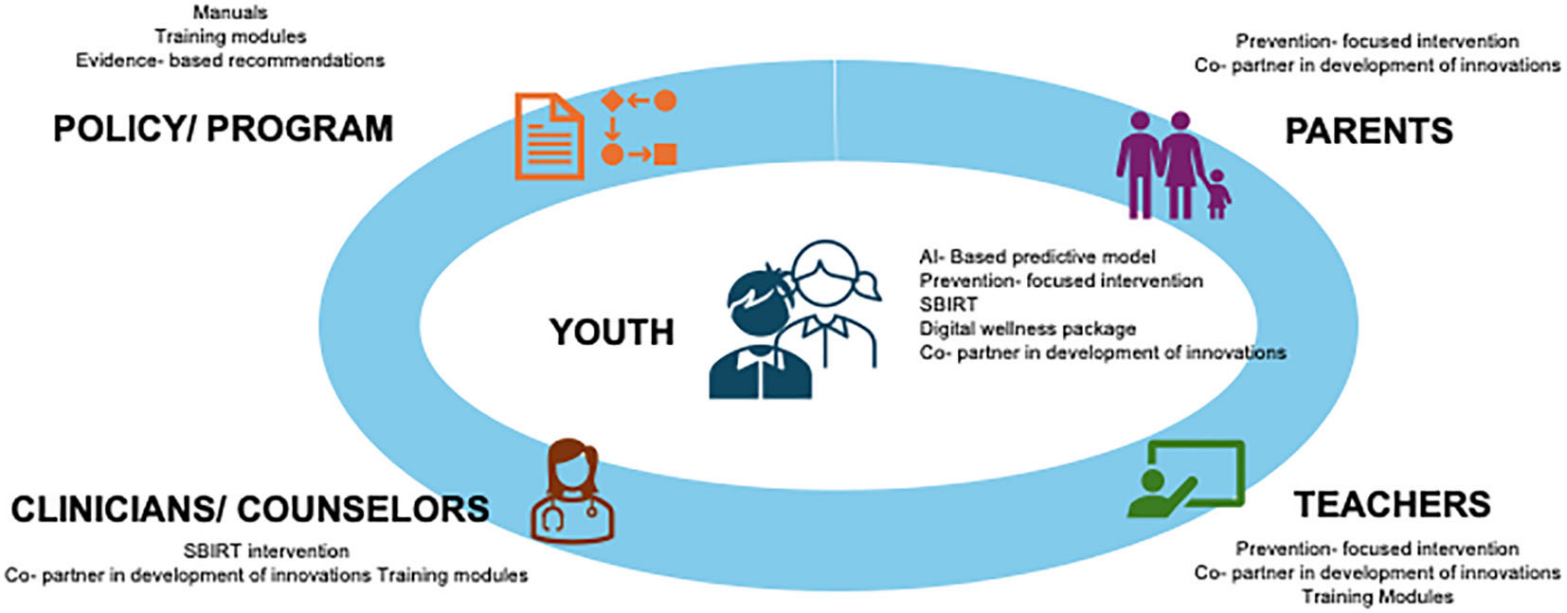
Conceptual schematic of various resources targeted at different stakeholders that shall be generated by Centre for Adcanved Research on Adddictive Behaviors (CAR-AB).

A snapshot of various resources targeted at different stakeholders that shall be generated has been presented in **Figure 4**.

## Discussion

CAR-AB is envisaged as a comprehensive, nationally coordinated initiatives to integrate AI-driven analytics, theory guided, and implementation-focused research in addressing behavioral addictions in India. Its transdisciplinary approach integrates psychiatry, psychology, nursing, technology, and education to generate actionable knowledge.

CAR-AB is expected to extend mental-health science beyond clinical settings into preventive domains. This aligns with the Global Mental Health Action Plan 2023–2030, emphasizing the promotion of mental well-being and prevention across the life course (47). The efforts of the CAR-AB are likely to contribute to the global initiatives from different research groups working in the field of behavioral addictions.

The CAR-AB research endeavor is anticipated to contribute to the multidimensional research in the field of behavioral addictions. The current research landscape of behavioral addiction in India is steadily expanding. India is a diverse country with variation in the socio-economic, linguistic, cultural, political, educational, and technological attributes and demographics of the population in question. The CAR-AB brings in opportunities for synergism, coordinated orchestration, and mutual influence in shaping the narrative of deliberating on contextually and culturally relevant research questions, and refining the modalities of treatment endeavors to answer descriptive and implementation research inquiries in the field of behavioral addictions (48).

While the initiative is based out of India, we envision the learning from the initiative to be of regional and global relevance. By focusing on behavioral addictions in low- and middle-income countries, CAR-AB will generate globally relevant evidence. Its frameworks can be adapted across South-East Asia and other regions facing similar digital-health challenges. The efforts of the CAR-AB are likely to contribute to the global initiatives from different research groups working in the field of behavioral addictions.

The findings from the studies conducted as part of CAR- AB shall be published in due course. The CAR-AB attempts to pool in data from several centers of India and focus on key research aspects of behavioral addictions. The CAR-AB examines a spectrum of research questions pertaining to behavioral addictions, encompassing understanding the determinants, developing scalable interventions, and field-testing these interventions. The research from CAR-AB would be generalizable to different regions of India. The horizon of behavioral addiction has also evolved over the last few years. While some of the state of the art evaluation methodologies (like linking with neuroimaging and genetics) have not been envisaged in the initial phase of the CAR-AB efforts, we remain open to expanding the scope of our work in future.

## Conclusion

This manuscript presents the conceptual framework, planned methodologies, and intended scope of the CAR-AB initiative. While grounded in existing evidence, the predictive models, interventions, and policy recommendations described herein are proposed and subject to empirical validation. Their effectiveness and public-health impact will be determined through future analyses and disseminated in subsequent publications. It intends to establish robust systems for early detection, prevention, and management of addictive behaviors. The initiative reflects a shift from exclusive clinical response to comprehensive, integrated and proactive public-health strategies. Its outputs will not only safeguard youth mental health but also contribute to global discourse on addictive behaviors and digital well-being. CAR-AB envisions “Digital Wellness for All” by promoting safe and healthy use of digital technology.

## Data Availability

The original contributions presented in the study are included in the article/supplementary material. Further inquiries can be directed to the corresponding author.
